# Combination of a permanent slow flow managed by the nurse and a rapid flow for bleeding management controlled by the doctor in underwater endoscopic submucosal dissection

**DOI:** 10.1055/a-2318-5558

**Published:** 2024-05-29

**Authors:** Auxane Chauveau, Soline Brun, Marina Cornelis, Muriel Deschamp, Laetitia Chevrot, Mikael Mochet, Mathieu Pioche

**Affiliations:** 1 36609 Gastroenterology, Hôpital Edouard Herriot, Lyon, France

Endoscopic submucosal dissection (ESD) is enjoying enormous growth, driven largely by all the technical innovations aimed at making it easier, safer, and faster 1 . Among the various means of facilitating submucosal exposure, floating the lesion to be resected in underwater dissection has become a precious technical aid in difficult cases, particularly when gravity is unfavorable for the situation 2 3 .

Underwater dissection does however pose a real technical problem when it comes to managing hemorrhage. A permanent low flow is necessary to maintain the floating effect of the lesion and to remove any dirt that gets into the cutting line, but this flow is not sufficient in the event of bleeding. Changing the flow rate during the procedure is not easy and generates stress when the bleeding is significant and the underwater field of vision turns red from the mixture of blood and water.

Our team of nurses came up with the idea of connecting two peristaltic pumps to the accessory channel of the endoscope via a T-fitting with two different foot pedals ( Fig. 1 and Fig. 2 ; Video 1 ): a low flow one that is used by the assistant to maintain a permanent low flow during dissection, and a maximum flow pedal that is put at the foot of the doctor as usual, for a higher flow in the event of bleeding, so that the area can be actively washed. In order to manage so many pedals without moving them and tangling up the wires, the IPEFIX device (Lyon, France) 4 helps us keep our workspace free of clutter.

**Fig. 1:**
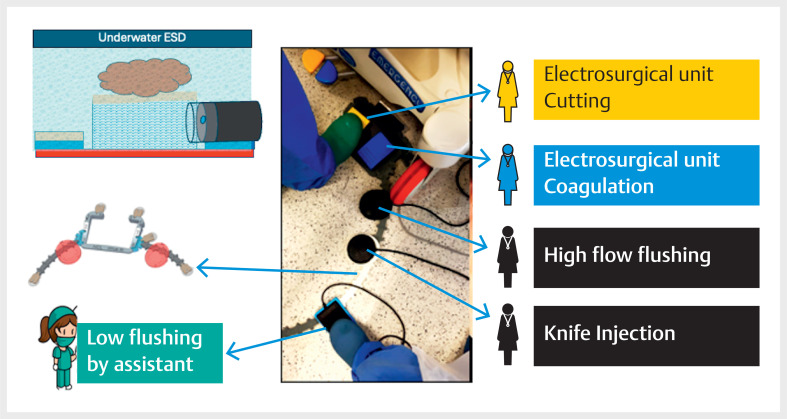
Schematic representation of the different foot pedals controlled by the IPEFIX, with pedals for both the nurse and the physician.

**Fig. 2:**
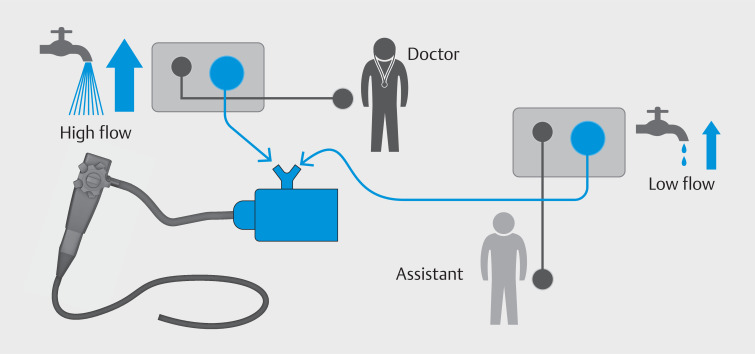
Schematic showing how the two pumps are connected.

Underwater endoscopic submucosal dissection is performed with double control using alternating low and high flow flushing controlled by the nurse and the physician, respectively.Video 1

Underwater ESD is an effective technique to deal with difficult procedures, and this technical trick helps greatly by offering the benefits of both low flow and high flow flushing in parallel, when it is necessary to deal with bleeding.

Endoscopy_UCTN_Code_TTT_1AQ_2AD_3AD

## Notes

Endoscopy E-Videos https://eref.thieme.de/e-videos
*E-Videos* is an open access online section of the journal *Endoscopy* , reporting on interesting cases and new techniques in gastroenterological endoscopy. All papers include a high-quality video and are published with a Creative Commons CC-BY license. Endoscopy E-Videos qualify for HINARI discounts and waivers and eligibility is automatically checked during the submission process. We grant 100% waivers to articles whose corresponding authors are based in Group A countries and 50% waivers to those who are based in Group B countries as classified by Research4Life (see: https://www.research4life.org/access/eligibility/ ). This section has its own submission website at https://mc.manuscriptcentral.com/e-videos .

## References

[1] Pioche M Masgnaux LJ Legros R Innovations for colonic endoscopic submucosal dissection: combination of the latest game changers Endoscopy 2024 56 242 243 10.1055/a-2191-5546 38417429 PMC10901623

[2] De Cristofaro E Masgnaux L-J Lupu A Treatment of a sessile serrated adenoma/polyp deeply invading the appendiceal orifice enabled by combined adaptive traction and underwater endoscopic submucosal dissection Endoscopy 2024 56 E215 E216 10.1055/a-2268-5673 38428918 PMC10907116

[3] Koyama Y Fukuzawa M Aikawa H Underwater endoscopic submucosal dissection for colorectal tumors decreases the incidence of post-electrocoagulation syndrome J Gastroenterol Hepatol 2023 38 1566 1575 10.1111/jgh.16259 37321649

[4] Yzet C Rivory J Wallenhorst T A 3D-printed pedal fixator for connecting different pedal-operated tools reduces the number of mistakes during endoscopic submucosal dissection Endosc Int Open 2023 11 E635 E640 37928772 10.1055/a-2095-0197 PMC10623429

